# Association of peripheral blood leukocyte *KIBRA* methylation with gastric cancer risk: a case–control study

**DOI:** 10.1002/cam4.1474

**Published:** 2018-04-16

**Authors:** Yan Zhang, Haibo Zhou, Hongxu Sun, Jie Chen, Di Huang, Xu Han, Xiyun Ren, Shangqun Lin, Qing Fan, Wenjing Tian, Yashuang Zhao

**Affiliations:** ^1^ Department of Epidemiology College of Public Health Harbin Medical University Harbin Heilongjiang Province China; ^2^ Xiangfang Center for Disease Control and Prevention Harbin Heilongjiang Province China

**Keywords:** Case–control study, gastric cancer, *KIBRA*, methylation, peripheral blood leukocyte

## Abstract

*KIBRA* was reported to be involved in various types of cancer and can be detected in blood. The purpose of this study was to investigate the relationship between the status of *KIBRA* methylation in peripheral blood leukocytes and gastric cancer (GC) risk. A case–control study was carried out to evaluate the association of blood cell‐derived *KIBRA* methylation with the risk of GC using methylation‐sensitive high‐resolution melting analysis. A total of 393 cases and 393 controls were detected, respectively. Compared with the subjects in the *KIBRA* negative methylation (NM) group, positive methylation (PM) subjects exhibited a 1.52‐fold (95% CI: 1.030–2.251, *P *=* *0.035) increased risk for GC. Stratified analyses demonstrated that the significant association of *KIBRA* methylation with GC risk existed in the older group (≥ 60 years; OR^a ^= 1.846, 95% CI: 1.037–3.287, *P *=* *0.037) and *Helicobacter pylori (H. pylori)* positive subjects (OR^a ^= 1.933, 95% CI: 1.103–3.386, *P *=* *0.021). Statistically significant combination effects between the environmental factors and *KIBRA* methylation on the GC risk were observed except for storing food under refrigeration. *KIBRA* methylation derived from blood cells and combinations thereof with environmental factors may be associated with the risk of GC.

## Introduction

Gastric cancer (GC) is one of the most commonplace digestive system malignant tumors 1. It is the third leading cause of cancer‐related deaths worldwide 2, 3. Although significant progress has been made in the treatment, it was still a major clinical challenge owing to its high incidence and poor prognosis of patients with advanced cancer 4. As is known to all, the occurrence of GC is affected by a variety of factors. Over the past few decades, multiple environmental factors underlying GC have been explored, including *H. pylori* infection 5.

In recent years, the relationship between epigenetic changes and GC has become a topic of interest 6. Epigenetic alterations are heritable changes in gene expression without an accompanying change in primary DNA sequence 7. DNA methylation is one of the most extensively studied epigenetic modifications, which occurs primarily in CpG islands within promoters. Hypermethylation of promoter CpG islands can induce the inappropriate silencing of tumor suppressor genes in the process of cancer initiation, progression, invasion, and metastasis 8, 9, 10, 11. This phenomenon was already demonstrated to be involved in the multistep progression of GC according to numerous studies 12, 13.

*KIBRA* (WW and C2 domain containing 1), an upstream component of the Hippo signaling pathway, was considered linked to cell proliferation and organ growth 14. It has been revealed that *KIBRA* functions as a tumor suppressor gene, and the expression of *KIBRA* was down‐regulated in acute lymphoblastic leukemia and clear cell renal cell carcinoma by promoter hypermethylation 15, 16. However, the role of *KIBRA* methylation in GC is largely unknown.

Previous studies suggested that the identification of DNA methylation alterations is not only in tissues from primary tumors but also in peripheral blood 17. Moreover, blood samples could capture more comprehensive information compared with tissues. Therefore, this case–control study was carried out to investigate the association of environmental factors, blood‐derived *KIBRA* methylation, and their interactions with the risk of GC. Furthermore, the relationship between environmental factors and *KIBRA* methylation was also explored.

## Materials and Methods

### Study subjects

A hospital‐based case–control study with 393 GC cases and 393 cancer‐free controls was carried out. Cases were newly diagnosed and pathologically confirmed GC patients of the Third Affiliated Hospital of Harbin Medical University in 2010 and 2012. Controls were ophthalmic and orthopedic patients recruited from the Second Affiliated Hospital of Harbin Medical University during the same period. All control subjects with a history of malignant tumors or gastrointestinal diseases were excluded. Every participant accepted a face‐to‐face interview using a structured questionnaire and donated 5 mL blood sample after giving informed consent. The questionnaire obtained information on demographic characteristics, dietary habits, lifestyle, and family history. “Long‐term drinking” was defined as consumed two or more alcoholic drinks per week for at least half a year. “Irregular diet” means have breakfast, lunch, and supper irregularly. This study protocol was approved by the Human Research and Ethics Committee of Harbin Medical University.

### *Helicobacter pylori* serologic tests

A serologic test for *H. pylori* IgG antibodies was performed in duplicate using an enzyme immunoassay kit (IBL, German). The sensitivity and specificity of the assay were greater than 95% stated by the manufacturer. Samples with 8 units/mL were considered as negative, 8–12 units/mL as equivocal, and 12 units/mL as positive.

### DNA extraction and bisulfite modification

Genomic DNA was extracted and bisulfite‐modified using QIAamp DNA Blood Mini Kit (Qiagen, Hilden, Germany) and EpiTect Fast DNA Bisulfite Kit (Qiagen), respectively. Nanodrop 2000 Spectrophotometer (Thermo Scientific) was used to quantify the DNA concentration. All procedures followed the manufacturers' instructions.

### Methylation‐sensitive high‐resolution melting (MS‐HRM) analysis

MS‐HRM assay was used to detect and analyze the status of *KIBRA* methylation. The assay was performed on the LightCycler^®^ 480 instrument (Roche, Mannheim, Germany) equipped with Gene Scanning software (version 2.0) in a 5 *μ*L volume reaction system, including 2.5 *μ*L LightCycler^®^ 480 High‐Resolution Melting Master Mix (Roche), 0.6 *μ*L MgCl_2_, 0.1 *μ*L forward primer, 0.1 *μ*L reverse primer, 0.5 *μ*L of sodium bisulfite‐modified DNA template, and 1.2 *μ*L PCR‐grade water.

Primers were designed using Primer Premier 5.0 software as follows, forward primer, 5′‐GCGTTCGGCGTTTCGTTT‐3′; reverse primer, 5′‐TCTCCGCTCCACCG CTCTA‐3′. Before the start of amplification, samples were preincubated for 10 min at 95°C. PCR reaction would proceed for 60 cycles and began with 10 sec DNA denaturation at 95°C. In order to minimize nonspecific amplification, a touchdown annealing temperature (58°C–48°C, 30 sec) was performed and further extension for 20 sec at 72°C. The amplified fragments were then melted from 67°C to 93°C with 30 signal acquisitions per degree (LightCycler480, Roche).

**Figure 1:**
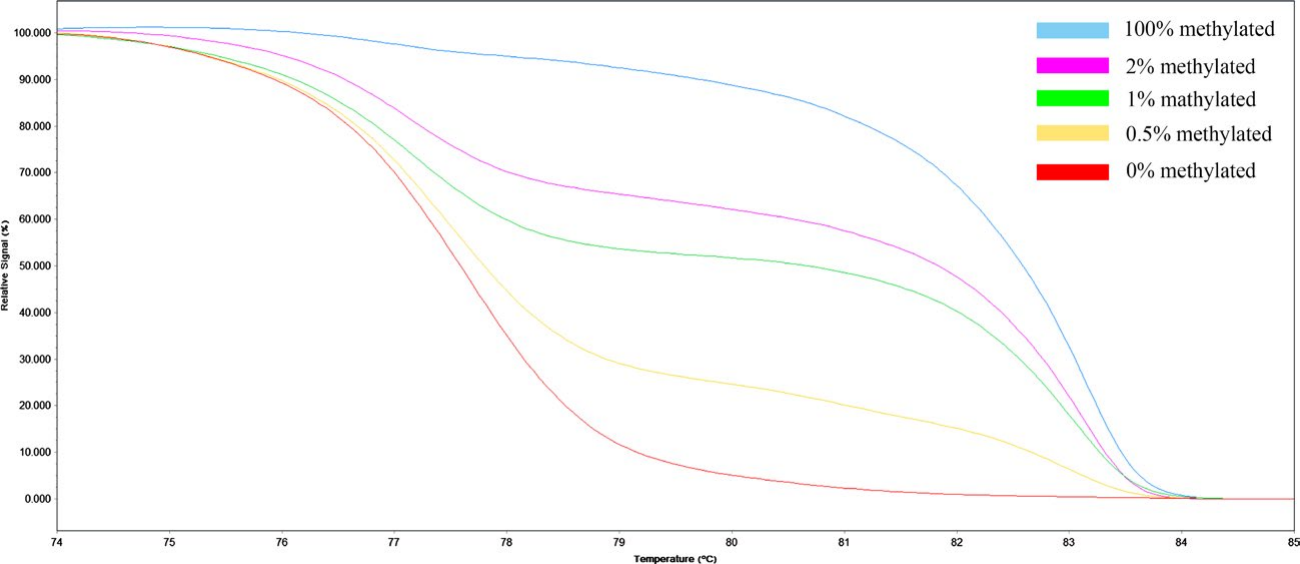
Profile of fluorescence obtained at the melting temperature for serial dilutions of methylated DNA (100%, 2%, 1%, 0.5%, and 0%) in *KIBRA* gene.

**Figure 2:**
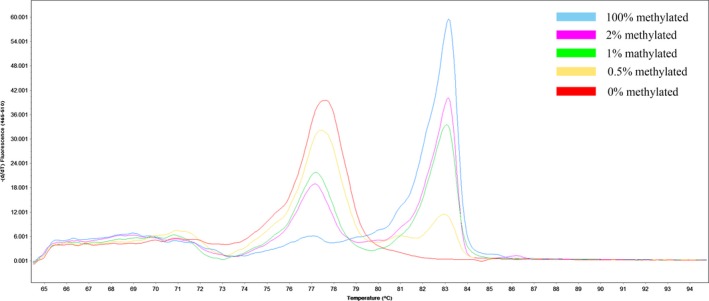
The melting peak of the *KIBRA* MS‐HRM assay.

A set of methylation standards, including 100%, 2%, 1%, 0.5%, and 0% methylated DNA, were constructed by mixing commercially available methylated (100% methylated) and unmethylated (0% methylated) human whole genomic DNA (Zymo Research). Figures 1 and 2 showed the profile of *KIBRA* standard curve and melting peak. The methylation status was determined by comparing the melting curves of samples with standards. Negative control (no‐template control) with PCR‐grade water was used in each batch and a second trial was conducted for equivocal results. Subjects were divided into negative methylation (NM) and positive methylation (PM) groups according to the cut‐off value of 0.5%.

### Statistical analysis

The chi‐square test (*χ* ^2^) was used for the categorical variables. Logistic regression analysis was applied to assess the effect of environmental factors and *KIBRA* methylation on the risk of GC, as well as the relationship between environmental factors and *KIBRA* methylation by odds ratios (ORs) and 95% confidence intervals (CIs). Gene‐environment interactions were estimated on a multiplicative scale with a product‐term coefficient using multivariable logistic regression. The combined effects were analyzed by crossover analysis. All statistical analyses were performed using SPSS version 19.0, *P* values < 0.05 were considered statistically significant.

## Results

### Characteristics of GC cases and controls

The basic demographic characteristics and clinicopathological features are listed in Table 1. Compared with the controls, despite the overall lower body mass index (BMI) and income level, the GC cases exhibited higher proportions of GC family history (*P *<* *0.05). According to the 6th American Joint Committee on Cancer TNM staging system for GC, 15% of cases were classified as stage I, 5.8% as stage II, 21.8% as stage III, and the majority of cases (57.4%) as stage IV.

**Table 1:** Characteristics of the study subjects

Variables	Cases (%)	Controls (%)	*P*
Age (years)			
<60	208 (51.1)	201 (51.1)	0.617
≥60	185 (48.9)	192 (48.9)	
Sex			
Male	298 (75.8)	297 (75.6)	0.950
Female	95 (24.2)	96 (24.4)	
Nation			
Han	371 (96.1)	371 (94.9)	0.409
Others	15 (3.9)	20 (5.1)	
BMI (kg/m^2^)			
<18.5	43 (11.3)	24 (6.2)	<0.001
18.5–22.9	185 (48.7)	133 (34.5)	
≥23	152 (40.0)	228 (59.2)	
Education			
Primary and below	113 (29.3)	113 (28.8)	0.996
Junior high school	144 (37.3)	147 (37.4)	
Senior middle school	80 (20.7)	84 (21.4)	
College and above	49 (12.7)	49 (12.5)	
Income			
<1000	134 (35.4)	178 (47.1)	0.001
≥1000	244 (64.6)	200 (52.9)	
Urban or rural			
Urban	238 (61)	238 (60.7)	0.929
Rural	152 (30)	154 (39.3)	
Family history			
Negative	331 (87.1)	378 (98.2)	<0.001
Positive	49 (12.9)	7 (1.8)	
Differentiation			
Low	263 (79.7)		
Middle	34 (10.3)		
High	33 (10)		
Clinical stage			
I	49 (15.0)		
II	19 (5.8)		
III	71 (21.8)		
IV	187 (57.4)		

### Relationships between environmental factors and GC risk

Univariate logistic regression analysis was used to evaluate relationships between environmental factors and GC risk, and the results were presented in Table S1. Fifteen factors were found to be significant. In the next step, backward variable conditional selection method (*P* values of 0.05 and 0.10 were specified as the thresholds for entry and removal of variables, respectively) was used for multivariate analysis. The results suggested that *H. pylori* infection, irregular diet, long‐term drinking, high‐salt diet, intake of overnight food (food being left overnight), and freshwater fish were positively associated with GC risk (*P *<* *0.05). Conversely, storing food under refrigeration, intake of vegetables (≥ 250 g/day), and garlic (≥ 1 time/week) showed a protective tendency on the risk of GC with ORs of 0.281, 0.236, and 0.221 (all *P* values < 0.05; additional data were given in Table S2).

### *KIBRA* methylation and GC risk

The percentage of *KIBRA* methylation was 83.7% and 77.0% in the cases and controls, respectively. Statistically significant association between the *KIBRA* PM and GC risk was observed (OR^c ^= 1.539, 95% CI: 1.075–2.202, *P = *0.018). Further analysis with adjustment for age, sex, BMI, income, and GC family history showed that the PM of *KIBRA* was still associated with GC (OR^a ^= 1.522, 95% CI: 1.030–2.251, *P = *0.035; Table 2).

**Table 2:** The relationship between *KIBRA* methylation and the risk of GC

Methylation	Controls (%)	Cases (%)	OR^c^ (95% CI)	*P*	OR^a^ (95% CI)	*P*
NM	88 (23.0)	64 (16.3)	1		1	
PM	294 (77.0)	329 (83.7)	1.539 (1.075, 2.202)	0.018	1.522 (1.030, 2.251)	0.035

OR^c^: Crude OR; OR^a^: Adjusted for age, sex, BMI, income, and family history of GC; PM, positive methylation; NM, negative methylation.

### Subgroup analysis

Stratified analysis was conducted in this study. Age‐stratified analysis showed that *KIBRA* PM conferred a significantly increased the risk of GC in the older group (≥ 60 years, OR^a^ *= *1.846, 95% CI: 1.037–3.287, *P = *0.037), while no significant difference was detected in the younger group (< 60 years). For the *H. pylori*‐infected individuals, the PM of *KIBRA* was positively associated with GC risk after adjusting for age, sex, BMI, income, and GC family history (OR^a^ *= *1.933, 95% CI: 1.103–3.386, *P = *0.021). However, no significant association was obtained in the negative *H. pylori* infection group (Table 3).

**Table 3:** Association of *KIBRA* methylation with the GC risk by age and *H. pylori* infection

Variables	NM (%)	PM (%)	OR^a^ (95% CI)	*P*
Age				
<60	82 (20.3)	321 (79.7)	1.258 (0.734, 2.156)	0.404
≥60	70 (18.8)	302 (81.2)	1.846 (1.037, 3.287)	0.037
*H.pylori* infection				
Negative	75 (20.4)	293 (79.6)	1.146 (0.651, 2.020)	0.637
Positive	77 (18.9)	330 (81.1)	1.933 (1.103, 3.386)	0.021

OR^a^: For an age‐stratified analysis, adjusted for sex, BMI, income, and family history of cancer; For a *H. pylori*‐stratified analysis, adjusted for age, sex, BMI, income, and family history of GC; PM, positive methylation; NM, negative methylation.

### Interactions of environmental factors and *KIBRA* methylation on the GC risk

As shown in Table 4, no significant interactions between the environmental factors and *KIBRA* methylation on the risk of GC were observed. However, there were significant combination effects between them. Long‐term drinking, irregular diet, high consumption of overnight food (≥ 1 time/week), freshwater fish (≥ 1 time/week), vegetables (≥ 250 g/day), and garlic (≥ 1 time/week) were associated with the GC risk in subjects independent of *KIBRA* methylation status.

**Table 4:** Combinations and interactions of *KIBRA* methylation and environmental factors on GC risk

Variables	NM	PM	Interaction	
	${\text{OR}}_{\mathrm{eg}}^{a}$ (95% CI)		${\text{OR}}_{i}^{a}$ (95% CI)	*P*
*H. pylori* infection				
Negative	1.000	1.141 (0.650, 2.001)	1.000	
Positive	1.169 (0.575, 2.376)	2.279 (1.309, 3.967)	1.709 (0.773, 3.779)	0.186
Irregular diet				
No	1.000	1.660 (1.039, 2.652)	1.000	
Yes	3.655 (1.599, 8.357)	5.290 (2.955, 9.470)	0.872 (0.345, 2.202)	0.772
High‐salt diet				
No	1.000	1.330 (0.799, 2.215)	1.000	
Yes	2.061 (0.992, 4.279)	3.232 (1.914, 5.459)	1.180 (0.525, 2.648)	0.689
Refrigerated food				
No	1.000	2.011 (0.871, 4.639)	1.000	
Yes	0.588 (0.248, 1.399)	0.758 (0.343, 1.676)	0.640 (0.247, 1.659)	0.359
Overnight food (times/week)				
<1	1.000	1.554 (0.710, 3.399)	1.000	
≥1	2.893 (1.250, 6.696)	3.693 (1.790, 7.618)	0.937 (0.378, 2.320)	0.888
Vegetables (g/day)				
<250	1.000	1.191 (0.357, 3.971)	1.000	
≥250	0.200 (0.065, 0.616)	0.330 (0.113, 0.965)	1.387 (0.386, 4.977)	0.616
Freshwater fish (times/week)				
<1	1.000	1.670 (1.036, 2.692)	1.000	
≥1	8.602 (3.256, 22.728)	7.395 (4.203, 13.010)	0.518 (0.181, 1.487)	0.222
Garlic (times/week)				
<1	1.000	1.465 (0.907, 2.367)	1.000	
≥1	0.191 (0.079, 0.460)	0.389 (0.228, 0.663)	1.381 (0.532, 3.589)	0.507
Long‐term drinking				
No	1.00	1.755 (0.979, 3.147)	1.000	
Yes	2.437 (1.149, 5.167)	3.240 (1.746, 6.012)	0.750 (0.338, 1.662)	0.478

${\text{OR}}_{\mathrm{eg}}^{a}$: Odds ratio generated by crossover analysis adjusted for age, sex, BMI, income, and family history of GC; ${\text{OR}}_{i}^{a}$: Odds ratio generated by multivariate logistic regression analysis adjusted for age, sex, BMI, income, and family history of GC; PM, positive methylation; NM, negative methylation.

### Effects of exposure to environmental factors on *KIBRA* methylation

According to the multivariable logistic regression analysis, storing food under refrigeration displayed a marginally significant negative association with *KIBRA* methylation in all subjects (*P *=* *0.049) and cases (*P *=* *0.033) but not in the controls, while high‐salt diet was associated with *KIBRA* methylation only in the cases (*P *=* *0.038; Table 5).

**Table 5:** Effect of exposure to environmental factors on *KIBRA* methylation

Variables	All subjects		Cases		Controls	
	OR^b^ (95% CI)	*P*	OR^a^ (95% CI)	*P*	OR^a^ (95% CI)	*P*
*H. pylori* infection						
Negative	1.000		1.000		1.000	
Positive	1.067 (0.737, 1.545)	0.732	1.193 (0.684, 2.080)	0.535	1.008 (0.609, 1.668)	0.975
Irregular diet						
Yes	1.000		1.000		1.000	
No	0.818 (0.524, 1.275)	0.374	0.751 (0.497, 1.656)	0.907	0.794 (0.397, 1.587)	0.514
High‐salt diet						
No	1.000		1.000		1.000	
Yes	1.458 (0.989, 2.147)	0.057	1.812 (1.034, 3.176)	0.038	1.165 (0.682, 1.992)	0.576
Refrigerated food						
No	1.000		1.000		1.000	
Yes	0.633 (0.401, 0.998)	0.049	0.503 (0.267, 0.947)	0.033	0.773 (0.396, 1.510)	0.452
Overnight food (times/week)						
<1	1.000		1.000		1.000	
≥1	1.260 (0.838, 1.897)	0.267	1.247 (0.635, 2.446)	0.521	1.311 (0.777, 2.213)	0.310
Vegetables (g/day)						
<250	1.000		1.000		1.000	
≥250	1.293 (0.744, 2.246)	0.362	1.501 (0.771, 2.924)	0.233	1.072 (0.378, 3.043)	0.896
Freshwater fish (times/week)						
<1	1.000		1.000		1.000	
≥1	1.176 (0.758, 1.847)	0.477	0.870 (0.497, 1.523)	0.625	2.039 (0.871, 4.772)	0.101
Garlic (times/week)						
≥1	1.000		1.000		1.000	
<1	1.192 (0.792, 1.794)	0.400	1.548 (0.736, 3.259)	0.250	1.066 (0.641, 1.771)	0.806
Long‐term drinking						
No	1.000		1.000		1.000	
Yes	1.015 (0.654, 1.576)	0.946	0.707 (0.343, 1.459)	0.349	1.272 (0.716, 2.259)	0.411

OR^a^: Adjusted for age, sex, BMI, income, and family history of GC; OR^b^: Adjusted for age, sex, BMI, income, GC family history, and case–control status.

## Discussion

Gastric cancer is considered as a systemic disease, and its epigenetic changes are not only confined to the lesion 18. During the development of GC, inflammation caused by carcinogenic factors can activate various immune responses, which lead to changes in leukocyte subpopulations, and further alter the DNA epigenetic signatures in early stage 19. It has been suggested that DNA methylation in peripheral blood leukocytes could be detected earlier and obtained more easily and also may capture more comprehensive information compared with tumor tissues 20, 21. These advantages of blood‐derived DNA methylation have led to many studies of its application in various cancers, including GC 22, 23. To date, the relationships between a list of tumor suppressor genes and the GC risk have been explored, but less is known about *KIBRA*.

In the current research, we attempted to investigate in peripheral blood leukocytes the *KIBRA* methylation status in GC cases and controls. Compared with NM subjects, PM subjects exhibited a 1.52‐fold increased risk for GC. Katrin Schelleckes et al. considered *KIBRA* methylation might interfere with binding of the transcription factor to DNA, down‐regulate its expression and impair Hippo signaling in renal cell carcinoma 16. However, whether the *KIBRA* methylation worked with the same mechanism in GC remains unclear and needs to be further explored.

It has been reported that age and *H. pylori* infection are variables that could influence the risk of GC and the DNA methylation alterations 24, 25, 26, thus stratification by these two variables was carried out in this study. Age‐stratified analysis demonstrated that *KIBRA* PM was associated with GC risk only in the older group (≥ 60 years). This was similar to the results of a monozygous twin study, which suggested that there was no significant methylation difference between twins during the early years of life, but in older twins, remarkable differences were observed 27. When the subjects were stratified according to the status of *H. pylori* infection, PM individuals had a higher GC risk in *H. pylori* positive group, while no association was found in the negative group. Considerable evidence has indicated that *H. pylori* infection‐triggered inflammation could induce DNA methylation, and methylation level would not return to baseline even though *H. pylori* was eradicated 28, 29. On the other hand, individuals exposed to *H. pylori* and *KIBRA* methylation simultaneously were more susceptible to GC compared with those who were unexposed or exposed to only one of them, which corroborated *H. pylori*‐stratified analysis result to some extent.

Several epidemiological studies have demonstrated that dietary factors and DNA methylation could independently and significantly influence the progression of GC 30, 31, while dietary factors play an important role not only in tumorigenesis but also in inducing DNA methylation 32, hence their interactions and combinations were explored in this study subsequently. Results showed that significant combinations were obviously observed between the dietary factors and *KIBRA* methylation on the GC risk, but no interactions were found. Among the studied dietary factors, high consumption of vegetables, freshwater fish, and garlic were considered to be protective factors while high‐salt diet, long‐term drinking, high consumption of overnight food and irregular diet performed as risk factors for GC in most previous studies 33, 34, 35, and these findings were replicated in this research except for freshwater fish. The result of this study indicated that high consumption of freshwater fish (≥ 1 time/week) increased the GC risk in subjects independent of *KIBRA* methylation status (OR = 7.395, 95% CI: 4.203–13.010; OR = 8.602, 95% CI: 3.256–22.728). Two possible reasons might be responsible for this unexpected result. First, freshwater fish raised in the industrial areas may have a high level of methyl mercury, polychlorinated dibenzofurans, organochlorine residues and other chemicals, and some of them have been proven to be highly carcinogenic 36, 37. Second, freshwater fish cooked by high‐temperature cooking methods like frying and grilling could induce the formation of heterocyclic amines, which might increase the susceptibility to cancer 38, 39.

Many researchers have reported that environmental factors affect the individual's susceptibility to GC, may be associated with altering the methylation status of genes 40, 41. Based on this point, we investigated the relationships between environmental factors and *KIBRA* methylation. In this study, storing food under refrigeration exhibited a trend toward decreased *KIBRA* methylation in all subjects and cases but not in the controls. Moreover, high‐salt diet was positively associated with *KIBRA* methylation only in the cases. Considering alteration of DNA methylation is a reversible process, making changes in diet might provide an opportunity to inhibit or reverse it and then prevent or delay the development of GC 42, 43. On the other hand, an appropriate diet could also reduce the *H. pylori* colonization or virulence levels, thereby decreasing the risk of GC 44.

In this research, respondents were asked about dietary habits 1 year prior to the interview. Due to the Chinese dietary diversity, memories about dietary habits may not be complete, which could lead to recall bias. In addition, the causality between *KIBRA* methylation and GC could not be implied in this retrospective study and additional prospective cohort studies are needed in the future.

## Conclusions

The results of this study indicated that *KIBRA* methylation derived from blood cells and combinations thereof with environmental factors may be associated with the risk of GC. These findings might have a public health value in indicating what people should avoid in their daily lives in terms of possible exposures that increase the GC risk.

## Conflict of Interest

None of the authors have any relevant conflict of interests to declare.

## Supporting information


**Table S1.** Univariate logistic analysis for the association of environmental factors with gastric cancer risk.
**Table S2.** Multivariate logistic analysis for the association of environmental factors with gastric cancer risk.
