# Supplementary material for: Association of peripheral blood leukocyte KIBRA methylation with gastric cancer risk: a case–control study
Source: Cancer Med. 2018 Apr 16;7(6):2682–90. doi: 10.1002/cam4.1474 (PMC6010778; doi:10.1002/cam4.1474)
Supplement: Supplementary file 1 — Table S1. Univariate logistic analysis for the association of environmental factors with gastric cancer risk. Table S2. Multivariate logistic analysis for the association of environmental factors with gastric cancer risk. [file CAM4-7-2682-s001.docx]

Table S1. Univariate logistic analysis for the association of environmental factors with gastric cancer risk.

| Variables | Cases (%) |  | Controls (%) |  | OR^c^ (95%CI) | *P* |  | OR^a^ (95%CI) | *p* |
| --- | --- | --- | --- | --- | --- | --- | --- | --- | --- |
|  |  |  |  |  |  |  |  |  |  |
| *H. pylori* infection |  |  |  |  |  |  |  |  |  |
| Negative | 161 (41.0) |  | 213 (54.2) |  | 1.000 |  |  | 1.000 |  |
| Positive | 232 (59.0) |  | 180 (45.8) |  | 1.705 (1.286,2.262) | ＜0.001 |  | 1.791 (1.314,2.441) | ＜0.001 |
| Irregular diet |  |  |  |  |  |  |  |  |  |
| No | 246 (63.9) |  | 330 (84.2) |  | 1.000 |  |  | 1.000 |  |
| Yes | 139 (36.1) |  | 62 (15.8) |  | 3.007 (2.137,4.233) | ＜0.001 |  | 3.174 (2.155,4.675) | ＜0.001 |
| High-salt diet |  |  |  |  |  |  |  |  |  |
| No | 175 (45.5) |  | 253 (64.5) |  | 1.000 |  |  | 1.000 |  |
| Yes | 210 (54.5) |  | 139 (35.5) |  | 2.184 (1.637,2.914) | ＜0.001 |  | 2.372 (1.729,3.255) | ＜0.001 |
| Hot food |  |  |  |  |  |  |  |  |  |
| No | 215 (56.0) |  | 208 (53.2) |  | 1.000 |  |  | 1.000 |  |
| Yes | 169 (44) |  | 183 (46.8) |  | 0.893 (0.673,1.186) | 0.435 |  | 0.799 (0.587,1.086) | 0.152 |
| Eating speed |  |  |  |  |  |  |  |  |  |
| Slow | 195 (50.6) |  | 222 (56.8) |  | 1.000 |  |  | 1.000 |  |
| Fast | 189 (49.1) |  | 169 (43.2) |  | 1.290 (0.974,1.709) | 0.076 |  | 1.585 (1.152,2.180) | 0.005 |
| Refrigerated food |  |  |  |  |  |  |  |  |  |
| No | 140 (36.4) |  | 74 (19.0) |  | 1.000 |  |  | 1.000 |  |
| Yes | 245 (63.6) |  | 316 (81.0) |  | 0.410 (0.295,0.569) | ＜0.001 |  | 0.391 (0.274,0.558) | ＜0.001 |
| Overnight food (times/week) |  |  |  |  |  |  |  |  |  |
| <1 | 73 (19.0) |  | 130 (33.4) |  | 1.000 |  |  | 1.000 |  |
| ≥1 | 311 (81.0) |  | 259 (66.6) |  | 2.138 (1.536,2.976) | ＜0.001 |  | 2.476 (1.717,3.569) | ＜0.001 |
| Vegetables (g/day) |  |  |  |  |  |  |  |  |  |
| <250 | 77 (20.1) |  | 24 (6.1) |  | 1.000 |  |  | 1.000 |  |
| ≥250 | 307 (79.9) |  | 368 (93.9) |  | 0.260 (0.160,0.421) | ＜0.001 |  | 0.269 (0.159,0.454) | ＜0.001 |
| Fruits (g/week) |  |  |  |  |  |  |  |  |  |
| <1000 | 236 (61.5) |  | 227 (57.9) |  | 1.000 |  |  | 1.000 |  |
| ≥1000 | 148 (38.5) |  | 165 (42.1) |  | 0.863 (0.647,1.150) | 0.313 |  | 0.866 (0.633,1.184) | 0.367 |
| Pork (g/week) |  |  |  |  |  |  |  |  |  |
| <250 | 142 (37.0) |  | 156 (40.8) |  | 1.000 |  |  | 1.000 |  |
| ≥250 | 242 (63.0) |  | 226 (59.2) |  | 1.176 (0.880,1.573) | 0.273 |  | 1.391 (1.003,1.928) | 0.048 |
| Poultry (g/week) |  |  |  |  |  |  |  |  |  |
| <0.5 | 335 (87.2) |  | 304 (79.8) |  | 1.000 |  |  | 1.000 |  |
| ≥0.5 | 49 (12.8) |  | 77 (20.2) |  | 0.577 (0.391,0.853) | 0.006 |  | 0.625 (0.408,0.958) | 0.031 |
| Freshwater fish (times/week) |  |  |  |  |  |  |  |  |  |
| <1 | 214 (56.3) |  | 336 (85.7) |  | 1.000 |  |  | 1.000 |  |
| ≥1 | 165 (43.4) |  | 56 (14.3) |  | 4.654 (3.286,6.592) | ＜0.001 |  | 5.021 (3.424,7.363) | ＜0.001 |
| Sea food (times/week) |  |  |  |  |  |  |  |  |  |
| <1 | 339 (89.2) |  | 349 (89.0) |  | 1.000 |  |  | 1.000 |  |
| ≥1 | 41 (10.8) |  | 43 (11.0) |  | 0.982 (0.624,1.544) | 0.936 |  | 0.912 (0.556,1.498) | 0.717 |
| Bean products (times/week) |  |  |  |  |  |  |  |  |  |
| ≤2 | 203 (53.1) |  | 165 (42.1) |  | 1.000 |  |  | 1.000 |  |
| >2 | 179 (46.9) |  | 227 (57.9) |  | 0.641 (0.483,0.851) | 0.002 |  | 0.608 (0.446,0.829) | 0.002 |
| Fried food (times/month) |  |  |  |  |  |  |  |  |  |
| <1 | 182 (47.4) |  | 221 (56.5) |  | 1.000 |  |  | 1.000 |  |
| ≥1 | 202 (52.6) |  | 170 (43.5) |  | 1.443 (1.087,1.915) | 0.011 |  | 1.469 (1.073,2.010) | 0.016 |
| Canned fruit (times/month) |  |  |  |  |  |  |  |  |  |
| <1 | 172 (83.1) |  | 267 (80.4) |  | 1.000 |  |  | 1.000 |  |
| ≥1 | 35 (16.9) |  | 65 (19.6) |  | 0.836 (0.531,1.315) | 0.438 |  | 0.643 (0.390,1.059) | 0.083 |
| Garlic (times/week) |  |  |  |  |  |  |  |  |  |
| <1 | 305 (79.2) |  | 203 (51.8) |  | 1.000 |  |  | 1.000 |  |
| ≥1 | 80 (20.8) |  | 189 (48.2) |  | 0.282 (0.205,0.386) | ＜0.001 |  | 0254 (0.179,0.362) | ＜0.001 |
| Tea |  |  |  |  |  |  |  |  |  |
| No | 275 (72.0) |  | 268 (68.4) |  | 1.000 |  |  | 1.000 |  |
| Yes | 107 (28.0) |  | 124 (31.6) |  | 0.841 (0.618,1.145) | 0.271 |  | 0.836 (0.591,1.183) | 0.311 |
| Smoking |  |  |  |  |  |  |  |  |  |
| No | 147 (38.4) |  | 188 (48.0) |  | 1.000 |  |  | 1.000 |  |
| Yes | 236 (61.6) |  | 204 (52.0) |  | 1.480 (1.112,1.969) | 0.007 |  | 1.601 (1.137,2.254) | 0.007 |
| Passive smoking |  |  |  |  |  |  |  |  |  |
| No | 180 (69.8) |  | 190 (70.9) |  | 1.000 |  |  | 1.000 |  |
| Yes | 78 (30.2) |  | 78 (29.8) |  | 1.056 (0.726,1.535) | 0.777 |  | 1.090 (0.726,1.635) | 0.679 |
| Long-term drinking |  |  |  |  |  |  |  |  |  |
| No | 161 (41.9) |  | 204 (52.3) |  | 1.000 |  |  | 1.000 |  |
| Yes | 223 (58.1) |  | 186 (47.7) |  | 1.519 (1.144,2.018) | 0.004 |  | 1.917 (1.323,2.778) | 0.001 |

OR^a^: Odds ratio generated by univariate logistic regression, adjusted for age, sex, BMI, income and family history of GC; OR^c^: Crude odds ratio

Table S2. Multivariate logistic analysis for the association of environmental factors with gastric cancer risk.

| Variables | *β*-coefficient | OR (95%CI) | *P* |
| --- | --- | --- | --- |
| Age | 0.037 | 1.038 (0.697,1.544) | 0.855 |
| Sex | 0.458 | 1.581 (0.924,2.704) | 0.094 |
| BMI |  |  | ＜0.001 |
| BMI(1) | -0.252 | 0.777 (0.363,1.667) | 0.518 |
| BMI(2) | -1.218 | 0.296 (0.137, 0.640) | 0.002 |
| Income | 0.707 | 2.027 (1.341, 3.064) | 0.001 |
| Family history | 2.722 | 15.218 (5.453, 42.465) | ＜0.001 |
| *H. pylori* infection | 0.536 | 1.710 (1.161, 2.517) | 0.007 |
| Irregular diet | 1.397 | 4.043 (2.511, 6.510) | ＜0.001 |
| High-salt diet | 0.777 | 2.176 (1.472, 3.217) | ＜0.001 |
| Refrigerated food | -1.270 | 0.281 (0.180,0.438) | ＜0.001 |
| Overnight food | 1.089 | 2.971 (1.854, 4.764) | ＜0.001 |
| Vegetables | -1.444 | 0.236 (0.125,0.447) | ＜0.001 |
| Freshwater fish | 1.883 | 6.571 (4.137,10.436) | ＜0.001 |
| Garlic | -1.510 | 0.221 (0.142,0.344) | ＜0.001 |
| Drinking | 0.583 | 1.792 (1.128,2.845) | 0.013 |

OR: Odds ratio; CI: Confidence interval.
